# Oral Bisphenol A Worsens Liver Immune-Metabolic and Mitochondrial Dysfunction Induced by High-Fat Diet in Adult Mice: Cross-Talk between Oxidative Stress and Inflammasome Pathway

**DOI:** 10.3390/antiox9121201

**Published:** 2020-11-30

**Authors:** Claudio Pirozzi, Adriano Lama, Chiara Annunziata, Gina Cavaliere, Clara Ruiz-Fernandez, Anna Monnolo, Federica Comella, Oreste Gualillo, Mariano Stornaiuolo, Maria Pina Mollica, Giuseppina Mattace Raso, Maria Carmela Ferrante, Rosaria Meli

**Affiliations:** 1 Department of Pharmacy, University of Naples Federico II, Via Domenico Montesano 49, 80131 Naples, Italy; claudio.pirozzi@unina.it (C.P.); adriano.lama@unina.it (A.L.); chiara.annunziata@unina.it (C.A.); clararf94@gmail.com (C.R.-F.); federica.comella@unina.it (F.C.); mariano.stornaiuolo@unina.it (M.S.); mattace@unina.it (G.M.R.); 2 Department of Biology, University of Naples Federico II, Cupa Nuova Cinthia 21-Edificio 7, 80126 Naples, Italy; gina.cavaliere@unina.it (G.C.); mpmollic@unina.it (M.P.M.); 3 SERGAS (Servizo Galego de Saude) and IDIS (Instituto de Investigación Sanitaria de Santiago), The NEIRID Lab (Neuroendocrine Interactions in Rheumatology and Inflammatory Diseases), Research Laboratory 9, Santiago University Clinical Hospital, 15706 Santiago de Compostela, Spain; oreste.gualillo@sergas.es; 4 Department of Veterinary Medicine and Animal Production, University of Naples Federico II, Via Delpino 1, 80137 Naples, Italy; anna.monnolo@unina.it

**Keywords:** obesity, reactive oxygen species, inflammatory cytokines, toll-like receptor-4, liver fibrosis, mitochondrial respiratory capacity, NLRP3 inflammasome signaling pathway

## Abstract

Lines of evidence have shown the embryogenic and transgenerational impact of bisphenol A (BPA), an endocrine-disrupting chemical, on immune-metabolic alterations, inflammation, and oxidative stress, while BPA toxic effects in adult obese mice are still overlooked. Here, we evaluate BPA’s worsening effect on several hepatic maladaptive processes associated to high-fat diet (HFD)-induced obesity in mice. After 12 weeks HFD feeding, C57Bl/6J male mice were exposed daily to BPA (50 μg/kg per os) along with HFD for 3 weeks. Glucose tolerance and lipid metabolism were examined in serum and/or liver. Hepatic oxidative damage (reactive oxygen species, malondialdehyde, antioxidant enzymes), and mitochondrial respiratory capacity were evaluated. Moreover, liver damage progression and inflammatory/immune response were determined by histological and molecular analysis. BPA amplified HFD-induced alteration of key factors involved in glucose and lipid metabolism, liver triglycerides accumulation, and worsened mitochondrial dysfunction by increasing oxidative stress and reducing antioxidant defense. The exacerbation by BPA of hepatic immune-metabolic dysfunction induced by HFD was shown by increased toll-like receptor-4 and its downstream pathways (i.e., NF-kB and NLRP3 inflammasome) amplifying inflammatory cytokine transcription and promoting fibrosis progression. This study evidences that BPA exposure represents an additional risk factor for the progression of fatty liver diseases strictly related to the cross-talk between oxidative stress and immune-metabolic impairment due to obesity.

## 1. Introduction

Bisphenol A (BPA) is considered one of the most widespread endocrine-disrupting chemicals (EDCs), substances affecting human health and impacting not only the endocrine system but also immune and metabolic functions [1,2]. Many lines of evidence have highlighted the link between impaired immunity and obesity and their relationship with EDC exposure [3,4].

An increase in oxidative stress-associated inflammation has been hypothesized to be one of the major mechanisms in the pathogenesis of obesity-related diseases [5]. As known, oxidative stress and inflammation are mutually dependent and connected. In fact, a rise in inflammatory cytokine levels drives a further increase in oxidative stress, sustaining a vicious cycle. In this regard, many toxic xenobiotics affect mitochondrial function and cause pro-oxidative conditions [6].

BPA, like several EDCs, can affect mitochondrial function, targets hepatic mitochondria [7], and predisposes to liver mitochondrial oxidative damage, altering the complex I activity of the mitochondrial electron transport chain [8], and ATP synthesis [9]. However, the involvement of mitochondrial dysfunction associated with BPA hepatotoxicity is disregarded and, even if critical periods of development have been analyzed, few pieces of evidence have been collected on the hepatotoxic effects of BPA on adult obese animals. However, BPA causes liver damage by the production of reactive oxygen species (ROS) and endoplasmic reticulum stress, and decreases fatty acid b-oxidation as shown in both in vitro and in vivo studies [10,11,12].

Increasing data have reported the adverse effects of BPA exposure, particularly during gestation and development [13,14]. BPA induces transgenerational obesity in rats [15] and following embryogenic exposure generates metabolic disturbances later in life, including diabetes and obesity both in offspring and in mothers themselves [16,17].

Perinatal exposure to BPA worsens hepatic alterations caused by a high-fat diet (HFD) in rat offspring [18]. Moreover, long-term exposure to HFD, simultaneously to BPA, induces insulin resistance (IR) in growing mice without affecting obesity and body adiposity [19]. Conversely, other authors have reported that perinatal BPA administration combined with HFD exacerbates dyslipidemia and obesity, and significant changes were observed in the expression of main factors involved in fatty acid metabolism [20].

Similar evidence has emerged in humans, where higher urinary concentrations of BPA were associated with morbidity in an adult population characterized by IR or central obesity [21]. In an exploratory study performed in healthy subjects, Stahlhut and co-authors [22] reported that oral administration of BPA (50 µg/kg) may induce an alteration of glucose-stimulated insulin response in humans. Notably, the dose of 50 µg/kg bw/day was considered the first safety reference dose (tolerable daily intake, TDI) for BPA [23] from 1988 by the Environmental Protection Agency and then adopted by Food and Drug Administration until 2015. More recently, the European Food Safety Authority (EFSA) has suggested a re-evaluation of TDI, indicating 4 µg/kg body weight daily as temporary TDI (t-TDI) (EFSA, 2017).

This study was focused on the evaluation of BPA’s exacerbating effect on liver immune-metabolic and mitochondrial dysfunction in adult HFD-fed obese mice, identifying the critical correlation and cross-talk between the oxidative stress and inflammasome pathway activation. We analyzed the impairment of glucose and lipid metabolism, the mitochondrial oxidative stress, the worsening of hepatic inflammatory damage in fibrosis, and the link between BPA and liver immunotoxicity related to obesity.

## 2. Materials and Methods

### 2.1. In Vivo Experimental Procedures

Male C57Bl/6J mice (20 ± 0.3 g/bw) (Charles River, Wilmington, MA, USA) at 6 weeks of age, were housed in stainless steel cages in a room kept at 22 ± 1 °C with a 12:12 h lights-dark cycle. All procedures involving the animals were carried out in conformity with international and national law and policies, including European Union (EU) Directive 2010/63/EU for animal experiments, Animal Research: Reporting of In Vivo Experiments (ARRIVE) guidelines 2.0, https://www.arriveguidelines.org/resources, the Basel Declaration, and the National Centre for the Replacement, Refinement and Reduction of Animals in Research (NC3Rs) concept, and were approved by the Institutional Committee on the Ethics of Animal Experiments (CSV) of the University of Naples Federico II and by the Italian Ministry of Health under protocol No. 371/2017-PR.

The standard chow diet had 17% fat without sucrose, while the HFD (Research Diets Inc, New Brunswick, NJ, USA) had 45% of energy derived from fat, 7% of sucrose. Standard chow diet (STD) and HFD contained 3.3 and 5.24 kcal/g, respectively. Six-week-old mice were randomly divided into three groups ensuring no differences in body weight mean (n = 15 animals for each group, 5–6 for each cage), as follows: (1) a control group receiving chow diet and vehicle per os (STD); (2) HFD group receiving vehicle; and (3) HFD group exposed daily to BPA (50 µg/kg per os, ≥99% purity, Sigma-Aldrich, Milan, Italy). Until 2015, the daily dose of BPA used in our experimental conditions was considered by the scientific community as the TDI for humans. The treatment started after 12 weeks of feeding with HFD, the time by which obesity was full-blown, and continued for 3 weeks. During the treatment, body weight and food intake were monitored weekly. Before sacrifice, bioelectrical impedance analysis (BIA) was performed to measure fat body composition using BIA 101 analyzer, modified for the mouse (Akern, Florence, Italy). Fat-free mass was calculated, and then fat mass content was determined as the difference between body weight and fat-free mass.

### 2.2. Biochemical, Hormone, and Hepatic Determinations

Blood obtained from STD, HFD, and HFD + BPA mice was collected by cardiac puncture and centrifuged at 2500 rpm at 4 °C for 12 min, and serum samples were stored at −80 °C for following biochemical and hormonal determinations. Alanine aminotransferase (ALT), alkaline phosphatase (ALP), triglycerides (TGL), and monocyte chemoattractant protein 1 (MCP1) were quantified by colorimetric enzymatic method using commercial kits (SGM Italia, Rome, Italy and Randox Laboratories ltd., Crumlin, UK). Serum leptin, adiponectin, lipopolysaccharide (LPS), and IL-10, (Thermo Scientific, Rockford, IL, USA), and fasting insulin (cat. no. EZRMI-13K; Millipore) concentrations were measured using commercially available ELISA kits. As an index of insulin resistance (IR), HOMA-IR (homeostasis model assessment) was calculated, using the formula (HOMA = fasting glucose (mmol/L) × fasting insulin (μU/mL)/22.5). Liver tissues were homogenized in saline solution and then centrifuged at 5000 rpm for 5 min. Supernatants were collected and centrifugated at 14,000 rpm at 4 °C for 15 min and triglycerides quantified (TGL Flex reagent cartridge, Siemens Healthcare GmbH, Erlangen, Germany).

### 2.3. Glucose and Pyruvate Tolerance Tests

At the end of the experimental period, oral glucose tolerance test (OGTT) was performed as previously described [24] on 16 h fasted animals (n = 7 each group), which received glucose (1 g/kg per os); glycemia was measured at 0, 30, 60, and 120 min. In another set of experiments, overnight fasted mice (n = 7 each group) were subjected to the pyruvate tolerance test (PTT). After blood glucose determination, animals were injected with pyruvate (2 g/kg i.p., Sigma-Aldrich, Milan, Italy), and glycemia was measured after 15, 30, 60, and 120 min. The area under the curve (AUC) was calculated as an integrated and cumulative measure of glycemia from time 0 up to 120 min for each animal. Glucose levels were measured by the glucometer One Touch Ultrasmart (Lifescan, Milpitas, CA, USA).

### 2.4. Measurements of Mitochondrial Oxidative Capacity and Enzyme Activity

Mitochondrial isolation and oxygen consumption were performed by high-resolution respirometry Hansatech oxygraph (Yellow Spring Instruments, Yellow Springs, OH, USA), as previously reported [25]. Oxygen consumption was measured in the presence of substrates and ADP (state 3) and in the presence of substrates alone (state 4), and their ratio (respiratory control ratio, RCR) was calculated. The specific activity of the carnitine palmitoyl-transferase (CPT) system, superoxide dismutase (SOD), and aconitase was spectrophotometrically measured, as previously described [26].

### 2.5. ROS Assay and Malondialdehyde Measurement

ROS and malondialdehyde (MDA) assays were performed as previously reported [27]. In ROS measurement, an equal volume of freshly prepared tissue homogenate was diluted in 100 mM potassium phosphate buffer (pH 7.4) and a final concentration of 5 μM dichloro-fluorescein diacetate (Sigma-Aldrich, Milan, Italy) in dimethyl sulfoxide for 15 min at 37 °C was added. The dye loaded samples were centrifuged at 12,500× *g* per 10 min at 4 °C. The pellet was mixed at ice-cold temperatures in 5 mL of 100 mM potassium phosphate buffer (pH 7.4) and then incubated for 60 min at 37 °C. The fluorescence was measured by the HTS-7000 Plus-plate-reader spectrofluorometer (Perkin Elmer, Wellesley, MA, USA) at 488 nm for excitation and 525 nm for emission wavelengths. ROS were quantified from the dichloro-fluorescein standard curve in dimethyl sulfoxide (0–1 mM). For MDA assay, tissues were homogenized in 1.15% KCl solution. A small amount of the homogenate (200 μL) was added to a reaction mixture containing 200 μL of 8.1% SDS, 1.5 mL of 20% acetic acid (pH 3.5), 1.5 mL of 0.8% thiobarbituric acid, and 600 μL of distilled water. The supernatant absorbance was spectrophotometrically measured at 550 nm and the concentration of MDA was expressed as micromoles of MDA normalized on mg of protein of tissue homogenate (µM/mg protein). A standard curve was prepared using MDA bis (dimethyl acetal) as the source of MDA.

### 2.6. Hepatic Histological Analysis

The liver was dissected and further fixed by immersion in 4% paraformaldehyde in PB overnight at 4 °C. Thus, the tissues were dehydrated in ethanol, cleared in xylene, and embedded in paraffin. Hematoxylin & eosin (H&E) staining was performed on paraffin-embedded sections (4 μm thick), as previously described [28] to assess liver morphology of all experimental groups. For the evaluation of fibrosis, alternate sections were stained with Mallory trichrome (MTC) by a standard procedure (Bio-Optica, Milan, Italy). Lobular inflammation, the appearance of ballooning degeneration, and necrosis were quantified as previously reported [24,29]. The grading system was adapted from the guidelines of the National Institutes of Health-sponsored Nonalcoholic Steatohepatitis Clinical Research Network (NASH CRN) [30]. The morphological analysis was performed in brightfield by a pathologist in a blinded manner. According to the NASH CRN system, the histological features were scored as follows: (i) ballooning: score 0—none; score 1—few balloon cells, score 2—many cells/prominent ballooning; (ii) lobular inflammation (as an overall assessment of all inflammatory foci): score 0—no foci; score 1—<2 foci per × 200 magnification field; score 2—2–4 foci per × 200 magnification field; score 3—>4 foci per × 200 magnification field; and (iii) necrosis: score 0—absent; score 1—present.

### 2.7. Protein Preparation and Western Blot Analysis

Livers were homogenized and total protein lysates were subjected to SDS-PAGE as previously described [28]. The blot was performed by Trans-Blot Turbo transfer system (Bio-Rad Laboratories, Segrate, Milan, Italy) at 240 mA for 60 min at room temperature. The obtained membrane filter was then blocked with 1X phosphate buffer solution (PBS) and 5% nonfat dried milk for 60 min at room temperature, and probed with anti-toll-like receptor (TLR) 4 rabbit polyclonal antibody (dilution 1:1000, Santa Cruz Biotechnology, Inc., Santa Cruz, CA, USA) and anti-NLRP3 rabbit monoclonal antibody (dilution 1:100, Cell Signaling Technology, Inc., Beverly, MA, USA). Western blot for β-actin (Sigma-Aldrich, Milan, Italy) was performed to ensure equal sample loading.

### 2.8. RNA Isolation and Real-Time PCR

Total RNA, isolated from the liver (n = at least 8 animals each group), was obtained by the extraction using TRIzol Reagent (Bio-Rad Laboratories) and following a specific RNA extraction kit (NucleoSpin^®^, MACHEREY-NAGEL GmbH & Co, Düren, Germany), according to the manufacturer’s instructions. cDNA was synthesized using High-Capacity cDNA Reverse Transcription Kit (Applied Biosystems, Waltham, MA, USA) as previously described [31], from 8 µg total RNA. PCRs were performed with a Bio-Rad CFX96 Connect Real-time PCR System instrument and software (Bio-Rad Laboratories, Segrate, Milan, Italy). The PCR conditions were previously reported [24]. Each sample contained 500 ng cDNA in 2X QuantiTech SYBRGreen PCR Master Mix and primers pairs to amplify *G6pc*, *Pck1*, *Fasn*, *Srebf1*, *Pparg*, *Tnfa*, *Ifng*, *Il6*, *Tgfb*, *Col1a1*, *Col3a1*, *Myd88*, NACHT, LRR and PYD domain-containing protein (*Nlrp3*), *Pycard*, *Casp1*, *Il1b*, *Nfkb1*, *Ccl2*, and *Itgax* (Qiagen, Hilden, Germany). The relative amount of each studied mRNA was normalized to *Actb* as a housekeeping gene, and the data were analyzed according to the 2^−ΔΔCt^ method.

### 2.9. Statistical Analysis

Data are presented as the mean ± SEM unless otherwise indicated. Differences among experimental groups were investigated through the one- or two-way (OGTT and PTT) analysis of variance (ANOVA) for multiple comparisons followed by Bonferroni’s post hoc test, using GraphPad Prism 8 (GraphPad Software, San Diego, CA, USA). Statistical significance was set at *p* < 0.05 in all the statistical analyses.

## 3. Results

### 3.1. Effect of BPA Exposure on Serum Biochemical Parameters and Adipokine Profile in HFD Mice

The increase in serum ALT, ALP, and triglycerides induced by HFD, was boosted by BPA (Table 1). Interestingly, BPA augmented systemic monocyte chemoattractant protein (MCP) 1 and portal LPS, and decreased IL-10 levels (Table 1), indicating an immunological involvement in BPA-induced detrimental effect on HFD-fed mice. The body weight gain of all groups was measured starting after 12 weeks HFD feeding and throughout the 3 weeks of treatment. In the HFD + BPA group, body weight gain and food intake did not change compared to HFD mice (data not shown), while a significant increase in fat mass was observed (STD = 2.823 ± 0.99 g; HFD = 10.68 ± 0.64 g, **** *p* < 0.0001 vs. STD; HFD + BPA = 13.91 ± 0.72 g, ^##^ *p* < 0.01 vs. HFD). Consistently, the adipokine (leptin and adiponectin) profile was altered by the HFD and further modified by BPA (Table 1), suggesting a worsening of metabolic alteration caused by an increase of leptinemia and leptin/adiponectin ratio.

### 3.2. BPA Exacerbates HFD-Induced Impairment of Glucose Homeostasis

**Figure 1:**
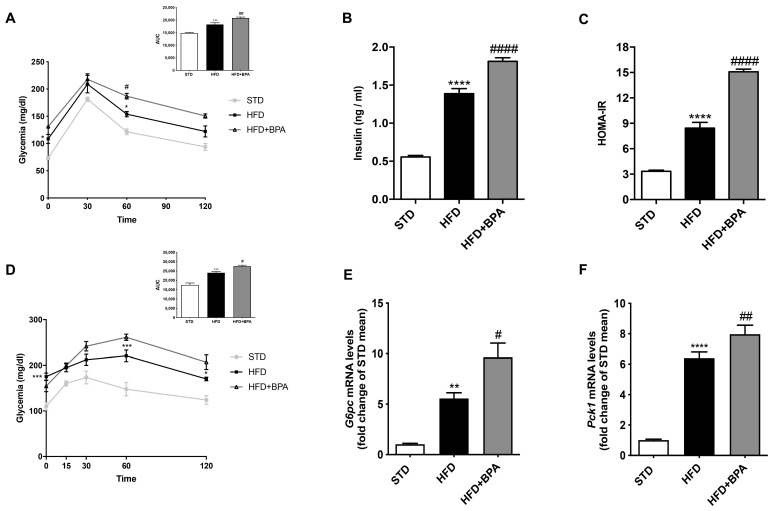
Bisphenol A (BPA) exacerbates glucose intolerance and gluconeogenesis already altered by high fat diet (HFD) feeding in mice. (**A**) Oral glucose tolerance test (OGTT) and (**D**) pyruvate tolerance test (PTT) were performed in all groups of animals (*n* = 7 each group). (**B**) Insulinemia and (**C**) homeostasis model assessment insulin resistance (HOMA-IR) index were determined; gene expression of (**E**) *G6pc* and (**F**) *Pck1* was measured in the liver of all animals by real-time PCR analysis. Data are presented as means ± SEM of all animals (n = 6 each group) (** *p* < 0.01, *** *p* < 0.001, and **** *p* < 0.0001 vs. STD; ^#^ *p* < 0.05, ^##^ *p* < 0.01, and ^####^ *p* < 0.0001 vs. HFD).

In another set of animals, an oral glucose tolerance test (OGTT) was performed at the end of the experimental time. As expected, HFD increased glycemia during OGTT compared to control; this alteration was amplified by BPA (Figure 1A). Consistently, BPA-treated HFD animals showed higher serum insulin levels than HFD (Figure 1B), resulting in a marked increase of the HOMA-IR index (Figure 1C). Intraperitoneal pyruvate administration in HFD + BPA animals induced a significant increase of glycemia compared to HFD mice (Figure 1D), indicating an alteration of de novo gluconeogenesis confirmed by the increase in *G6pase* and *Pck1* gene expression in the liver (Figure 1E,F).

### 3.3. BPA Exposure Worsens Liver Inflammation in HFD-Fed Mice

**Figure 2:**
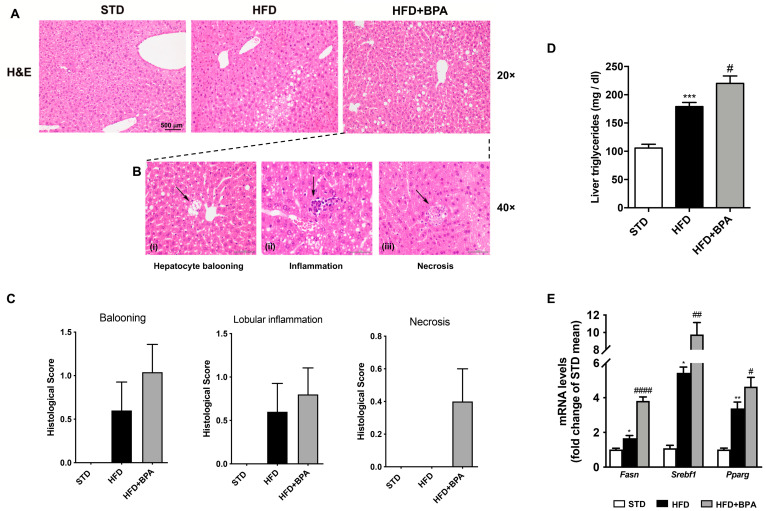
BPA exposure worsens hepatic steatosis and inflammation in HFD-fed mice. (**A**) H&E staining was performed on the liver from all groups of animals (magnification 20×) (*n* = 4 each group). (**B**) (i) The appearance of the ballooning degeneration, (ii) the inflammatory and (iii) necrotic lesions in the liver from HFD + BPA, and (**C**) the histological score for all three groups were shown. (**D**) Hepatic triglycerides and mRNA expression of (**E**) *Fasn*, *Srebf1*, and *Pparg* were determined. Data are presented as means ± SEM of all animals (n = 6 each group) (* *p* < 0.05 vs. STD, ** *p* < 0.01, *** *p* < 0.001, ^#^ *p* < 0.05, ^##^ *p* < 0.01, and ^####^ *p* < 0.0001 vs. HFD).

The H&E staining of hepatic tissue from HFD mice evidenced a slight and localized steatosis (Zone 1 steatosis) (Figure 2A). BPA exposure aggravated the steatotic pattern, spreading the azonal steatosis, and inducing the appearance of the ballooning degeneration (i), the inflammatory (ii), and necrotic lesions (iii) (Figure 2B). The histological score among all three groups was shown in Figure 2C. Consistently, we showed the marked increase by BPA of hepatic triglycerides (Figure 2D) and the mRNA expression of different genes involved in fatty acid synthase and/or accumulation in the liver, such as *Fasn*, *Srebf1*, *Pparg* (Figure 2E), already altered by HFD.

### 3.4. BPA Worsens Liver Mitochondrial Efficiency and Oxidative Stress Induced by HFD

**Figure 3:**
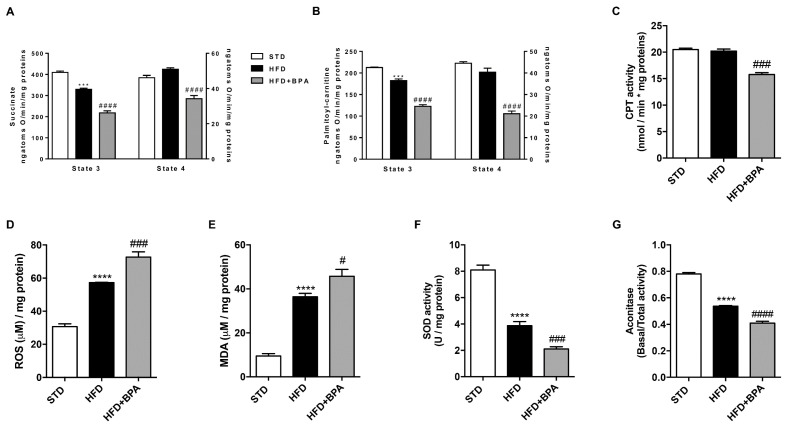
BPA effect on liver mitochondrial dysfunction and oxidative stress induced by HFD. (**A**) Mitochondrial state 3 and 4 respiration and (**B**) oxygen consumption linked to fatty acid oxidation were measured in the liver from all experimental groups using succinate or palmitoyl-carnitine as substrates, respectively. (**C**) The mitochondrial carnitine palmitoyl-transferase (CPT) activity in the liver of BPA-treated mice was determined. BPA worsening effect on HFD-induced (**D**) hepatic reactive oxygen species (ROS), and (**E**) malondialdehyde (MDA) levels was shown. Hepatic mitochondrial (**F**) superoxide dismutase (SOD) and (**G**) aconitase activity was spectrophotometrically measured. Data are presented as means ± SEM of all animals (*n* = 6 each group) (*** *p* < 0.001, and **** *p* < 0.0001 vs. STD; ^#^ *p* < 0.05, ^###^ *p* < 0.001, and ^####^ *p* < 0.0001 vs. HFD).

In hepatic mitochondria, the evaluation of oxygen consumption using succinate as substrate, was performed (Figure 3A). State 3 respiration was reduced in HFD-fed animals compared to STD mice and it was further damaged by BPA (Figure 3A). To examine mitochondrial fatty acid oxidation, state 3 respiration was also evaluated using palmitoyl-carnitine as a substrate (Figure 3B). BPA markedly reduced the oxygen consumption compared with no treated HFD mice in both determinations. Variations were observed in mitochondrial state 4 respiration between HFD and HFD + BPA groups using both succinate and palmitoyl-carnitine as a substrate (Figure 3A,B). High quality of mitochondrial preparations was ensured by the measurement of high respiratory control ratio in all groups (data not shown). According to these findings, we demonstrated the reduction of mitochondrial CPT activity (Figure 3C) in the liver of BPA-treated mice, confirming the worsening of lipid metabolism related to the impairment of fatty acid β-oxidation. The deep alteration of hepatic mitochondrial respiratory capacity was linked to BPA-intensified oxidative stress, shown by increasing hepatic ROS production and MDA levels, already induced by HFD (Figure 3D,E). Moreover, BPA limited the antioxidant defense, dampening the mitochondrial activity of SOD and aconitase enzymes (Figure 3F,G).

### 3.5. BPA Amplifies Immune/Inflammatory Response and Induces NLRP3 Inflammasome Activation in the Liver of HFD Mice

**Figure 4:**
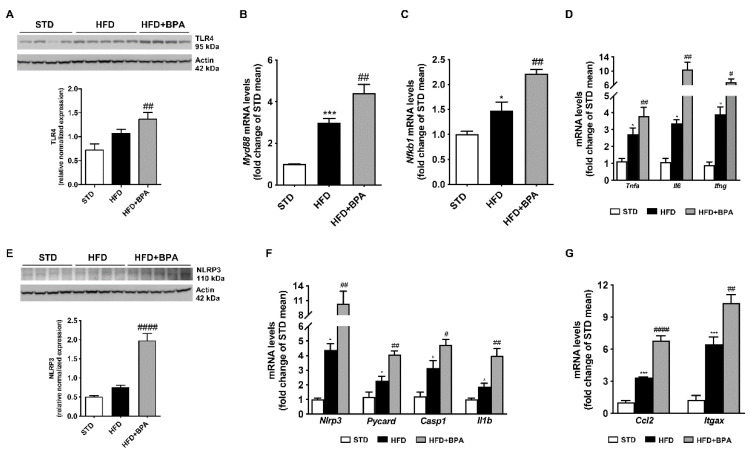
BPA amplifies immune response and cytokine transcription and induces inflammasome activation in the liver of HFD mice. Protein expression of (**A**) TLR4 and mRNA levels of (**B**) *Myd88*, and (**C**) *Nfkb1* were determined in the liver from all mice. mRNA transcript of (**D**) *Tnfa*, *Il6*, and *Ifng* was measured. The activation of hepatic inflammasome complexes by BPA was shown by the marked increase of (**E**) NLRP3 protein expression, and the upregulation of (**F**) *Nlrp3*, *Casp1*, *Pycard*, and *Il1b* transcripts, evaluated by Western blot and real-time PCR, respectively. Cropped Western blots for TLR4 and NLRP3 were shown. The increase of chemokine-guided monocyte recruitment induced by BPA was demonstrated by the evaluation of mRNA transcription of (**G**) *Ccl2* and *Itgax*. Data are presented as means ± SEM of all animals (*n* = 6 each group) (* *p* < 0.05, and *** *p* < 0.001 vs. STD; ^#^ *p* < 0.05, ^##^ *p* < 0.01, and ^####^ *p* < 0.0001 vs. HFD).

As shown in Figure 4, BPA exacerbated hepatic inflammation and immune response induced by HFD. BPA significantly induced TLR4 protein expression and its downstream gene *Myd88* (Figure 4A,B), as well as *Nfkb1* mRNAs and the transcription of pro-inflammatory cytokines, including *Tnfa*, *Il6*, and *Ifng* (Figure 4C,D). Notably, BPA toxic effect was linked to the inflammasome pathway activation, as shown by the marked induction of NLRP3 expression, and the following up-regulation of the adaptor ASC (*Pycard*), and caspase 1 (*Casp1*) mRNAs, and the strictly related IL-1β (*Il1b*) transcription (Figure 4E,F), already altered by HFD. Notably, BPA induced the hepatic monocyte and macrophage recruitment, as shown by the increase of MCP1 (*Ccl2*) and macrophage recruitment factor Cd11c (*Itgax*) mRNAs (Figure 4G).

### 3.6. BPA Exposure Promotes Liver Fibrosis Progression in HFD-Fed Mice

**Figure 5:**
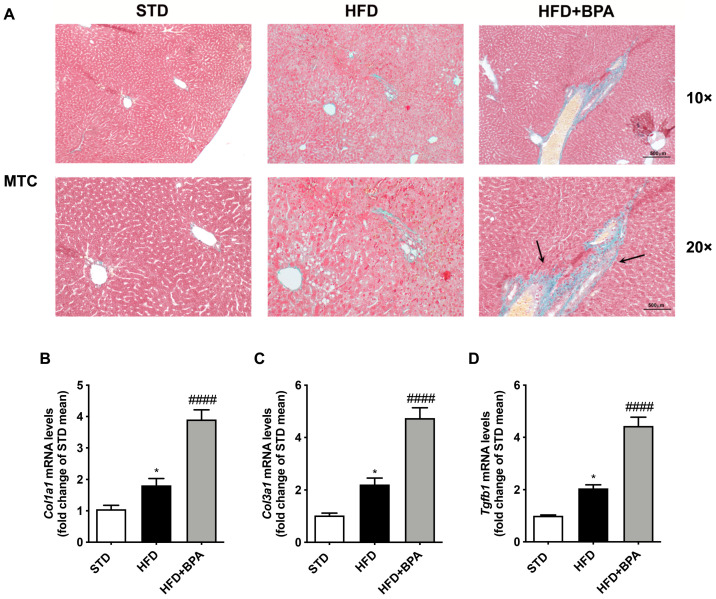
BPA exposure promotes liver fibrosis progression in HFD-fed mice. (**A**) Mallory trichrome (MTC) staining was assessed to determine the fibrotic lesions induced by BPA on the liver from HFD mice (*n* = 4 each group). Gene expression of pro-fibrotic (**B**–**D**) *Col1a1*, *Col3a1*, and *Tgfb* was determined by real-time PCR analysis. Data are presented as means ± SEM of all animals (*n* = 6 each group) (* *p* < 0.05 vs. STD; ^####^ *p* < 0.0001 vs. HFD).

To better define the effect of BPA on the progression of fatty liver diseases associated to HFD feeding, we assessed MTC staining (Figure 5A). The liver from HFD + BPA mice showed an extended and irregular collagen deposition around the vessel (Figure 5A). Accordingly, BPA exposure markedly induced the mRNA expression of pro-fibrotic factors, such as *Col1A1*, *Col3a1*, and *Tgfb*, slightly altered by HFD (Figure 5B–D).

## 4. Discussion

In this study we demonstrate that a sub-chronic BPA exposure in HFD-induced obese mice worsens most of the features related to obesity in adulthood. We investigate the mechanisms underpinning immune-metabolic impairment focusing on oxidative stress and hepatic mitochondrial dysfunction related to inflammasome activation causing the progression of tissue damage.

Previous evidence describes BPA, similarly to other EDCs, as an inducer of epigenetic transgenerational inheritance of obesity [1]. Embryonic, perinatal, and early life exposure to BPA generates metabolic alterations later in life predisposing to obesity and diabetes [1,2], evidencing its role in the etiology of metabolic disorders. Here, we have determined the mechanisms underlined to liver toxic effect of BPA in adult obese mice, regardless of perinatal exposure, since obesity-related disorders are associated with BPA exposure in both children and adults [32,33]. Moreover, in healthy subjects, a single oral administration of BPA (50 μg/kg) causes an altered insulin response to glucose stimulation [22]. Generally, the toxic effects of the xenobiotics depend on concentration/dose and time of exposure. In preclinical in vivo and in vitro studies planned to explore the molecular mechanisms of EDCs, high doses or concentrations are usually preferred [34,35]. In our experimental condition, we chose 50 μg/kg based on literature data considering the short time of mice exposure to BPA (3 weeks).

Here, BPA did not modify body weight and food intake but significantly increased fat mass, as well as serum hepatic enzymes and triglycerides. Moreover, BPA altered adipokine and hormone profile as evidenced by the increased leptin/adiponectin ratio and HOMA-IR. As known, leptin and adiponectin are inversely involved in glucose and lipid metabolism, through AMP-activated protein kinase (AMPK) activation [36]. Here, BPA exposure of obese mice markedly reduces oral glucose and pyruvate tolerance and increases key enzymes or mediators involved in gluconeogenesis (i.e., G6Pase and PCK1), de novo lipogenesis (i.e., fatty acid synthase or FAS, and sterol regulatory element-binding protein-1c or SREBP-1c), and triglycerides accumulation in the liver. Previous data have demonstrated that BPA exposure in offspring fed with HFD reduced peroxisome proliferator-activated receptor (PPAR)-α and CPT1 expression, leading to hepatic lipid accumulation through the inhibition of fatty acid oxidation [18]. Consistently, we observe a reduction of mitochondrial CPT activity in the liver from BPA-treated adult obese mice, which causes the accumulation of toxic lipid-derived metabolites in hepatocytes. Excessive free fatty acid amounts in the liver can activate the signaling pathways that promote oxidation, inflammation, and fibrosis [37].

To date, no evidence regarding BPA toxicity on hepatic mitochondria in adult obese animals is available. Our previous studies showed that HFD causes the alteration of liver mitochondrial function and dynamics in mice, also reducing the activity of antioxidant scavengers [24,38]. Here, we prove the worsening effect of BPA on HFD-induced hepatic mitochondrial damage. Indeed, BPA further reduced HFD-impaired respiratory capacity, enhancing ROS and MDA levels, and inhibiting the activity of detoxifying enzymes (i.e., SOD and aconitase); more interestingly, BPA also dampened CPT activity, leading to an increase of hepatic ectopic lipid storage, as demonstrated by triglycerides’ accumulation. Consistent with the BPA-induced alteration of redox balance, Khan et al. [7] observed a decrease in glutathione (GSH) levels and an increase of superoxides in BPA-treated rats.

Our data agree with previous studies reporting that BPA interferes with mitochondrial functions in the liver and other tissues [7,39] compromising the respiratory chain, reducing OXPHOS capacity, and increasing oxidative stress [34,39]. Moreover, the alteration of mitochondrial bioenergetics, dynamics, and apoptosis by BPA has been recently demonstrated [7,40]. In three-week-old offspring, perinatal exposure to BPA decreases mitochondrial respiratory complex activity and modifies the expression of genes involved in fatty acid metabolism, without alteration of liver morphology and function [41].

In our experimental condition, BPA increases systemic inflammation induced by fat overnutrition. Indeed, higher levels of LPS and MCP1, and the reduction of anti-inflammatory IL-10 were observed in serum. LPS is one of the most crucial factors contributing to low-grade inflammation, also called “metainflammation”, associated with HFD feeding, responsible for the induction of inflammatory cytokines by immune cells and adipocytes [38].

Several lines of evidence have previously demonstrated BPA’s capability of inducing the dysregulation of cytokines, hepatocyte apoptosis, and oxidative stress in the liver [11,42]. Moreover, Moon et al. [19] showed that long-term simultaneous exposure to HFD and BPA induced the alteration of glucose homeostasis and insulin sensitivity in growing mice without modifying adiponectin and inflammatory cytokines. On the other hand, adiponectin level was suppressed in prenatal BPA-treated animals even if they did not show steatosis features [17]. Recently, clinical evidence on the adult male population revealed the association among BPA plasma levels and inflammatory markers, visceral obesity, and IR [43].

During lipid overnutrition, circulating free fatty acids, whose levels are commonly increased in obesity, accumulate in the liver as fat storage, and at the same time, in concert with LPS, trigger TLR4/NF-κB pathway [44]. Therefore, as a consequence, the massive production of pro-inflammatory cytokines in hepatic cells (i.e., hepatocytes and Kupffer cells) leads to the alteration of the leptin/adiponectin ratio and promotes the conversion of steatosis into steatohepatitis causing liver damage progression [45]. Consistently, the deleterious effect of BPA results in the exacerbated activation of innate immune response as evidenced by the further increase of hepatic TLR4, its adaptor protein MyD88, as well as the downstream target NF-κB and excessive cytokine production. It is known that in nonalcoholic fatty liver disease (NAFLD) patients as well as animal models of NAFLD, NF-kB activation is observed in liver cells, including hepatocytes, hepatic stellate cells, and Kupffer cells [46,47]. Among these, hepatocytes respond minimally to TLR ligands suggesting that also other mediators can activate NF-kB in hepatocytes. On the other hand, these TLR ligands directly activate NF-kB in Kupffer cells, sustaining the vicious cycle of the inflammatory response.

Therefore, our data demonstrate that BPA exposure aggravates liver damage induced by HFD, not only causing azonal steatosis but also increasing steatohepatitis, accelerating the fibrotic process proven by marked collagen deposition and pro-fibrotic factors expression. Indeed, steatosis progression to steatohepatitis and fibrosis originates from the uncontrolled increase of oxidative damage and inflammatory cascade [48] and then is sustained by the activation of NLRP3 inflammasome which promotes Casp1-dependent IL-1β production [49]. Indeed, the pivotal role of inflammasome activation in NAFLD progression has been shown in patients with NASH [50] and knockout mouse models [51]. To date, little evidence is available regarding BPA activation of inflammasome complexes, evidenced only in in vitro systems, in the macrophages [52] or myeloid cells [53]. Notably, our data show that in obese mice, BPA induces hepatic NLRP3 inflammasome pathway, increasing the adaptor ASC, Casp-1, and amplifying IL-1β production. Casp-1 and ASC, as well as inflammasome complexes, have been found as master regulators of IL-1β activation or signaling, which is required for the development of liver steatosis, inflammation, and damage [54]. Furthermore, in our experimental condition, BPA-induced inflammasome activation and IL-1β production is related to the monocyte/macrophage recruitment in the liver (i.e., increased MCP1 and Cd11c mRNA), amplifying the vicious cycle of immune/pro-inflammatory process.

However, we cannot exclude the involvement of other mechanisms underlying BPA liver toxicity. Among these, oxidative stress caused by BPA exposure can induce epigenetic changes [34], including DNA methylation that modulates metabolic/endocrine processes and diseases [55,56,57]. According to these findings, our preliminary data show the alteration of global DNA hypermethylation by BPA in the liver from obese mice, demonstrating an exacerbation of epigenetic changes induced by HFD (Figure S1, Supplementary Materials). Further investigations will be needed to clarify the link between BPA, oxidative stress, and epigenetic modifications in obese mice, especially in terms of likely methyl donors (i.e., methionine and S-Adenosyl Methionine or SAM) [58].

## 5. Conclusions

In summary, BPA aggravates liver immune-metabolic and mitochondrial dysfunction in adult obese mice magnifying the cross-talk among hepatic oxidative stress, cytokine network, and fibrosis progression, and highlighting the critical role of inflammasome activation. Therefore, our data point out that BPA exposure represents an additional risk factor for the progression of fatty liver diseases and the other pathological features strictly related to obesity in adulthood.

## Figures and Tables

**Table 1:** Effect of BPA on serum biochemical parameters and adipokine profile in HFD mice.

Serum Parameters	STD	HFD	HFD + BPA
ALT (U/L)	65.0 ± 2.12	139.2 ± 4.03 ****	183.2 ± 3.16 ^####^
ALP (U/L)	155.0 ± 3.44	265.6 ± 4.58 ****	288.2 ± 6.41 ^#^
Triglycerides (mg/dL)	114.6 ± 5.391	218.6 ± 6.87 ****	258.3 ± 6.86 ^##^
MCP-1 (pg/mL)	28.50 ± 1.28	61.0 ± 2.00 ****	74.6 ± 3.55 ^##^
LPS (U/mL)	0.62 ± 0.02	1.53 ± 0.02 ****	1.84 ± 0.04 ^##^
IL-10 (ng/mL)	0.044 ± 0.002	0.034 ± 0.003 *	0.019 ± 0.001 ^##^
Leptin (ng/mL)	1.08 ± 0.04	12.88 ± 0.36 ****	15.65 ± 0.42 ^###^
Adiponectin (mg/mL)	2.87 ± 0.09	1.26 ± 0.06 ****	1.07 ± 0.07 ****
Lep/Adipo ratio	0.38 ± 0.02	9.63 ± 0.08 ****	15.59 ± 0.81 ^####^

Data are presented as mean ± SEM of all animals from different groups (*n* = 6–8) (* *p* < 0.05, **** *p* < 0.0001 vs. STD; ^#^ *p* < 0.05, ^##^ *p* < 0.01, ^###^ *p* < 0.001, ^####^ *p* < 0.0001 vs. HFD. Abbreviations: ALT, alanine aminotransferase; ALP, alkaline phosphatase; MCP-1, monocyte chemoattractant protein 1; LPS, lipopolysaccharide; IL-10, interleukin-10.

## Supplementary Materials

The following are available online at https://www.mdpi.com/2076-3921/9/12/1201/s1, Figure S1: BPA increases hepatic global DNA methylation induced by HFD.
