# Supplementary material for: Oral Bisphenol A Worsens Liver Immune-Metabolic and Mitochondrial Dysfunction Induced by High-Fat Diet in Adult Mice: Cross-Talk between Oxidative Stress and Inflammasome Pathway
Source: Antioxidants (Basel). 2020 Nov 30;9(12):1201. doi: 10.3390/antiox9121201 (PMC7760359; doi:10.3390/antiox9121201)
Supplement: Supplementary file 1 [file antioxidants-09-01201-s001.zip › antioxidants-1010380-suppl-final check/Supplementary Materials.docx]

**Supplementary Materials –*Antioxidants***

Oral Bisphenol A Worsens Liver Immune-Metabolic and Mitochondrial Dysfunction Induced by High-Fat Diet in Adult Mice: Cross-Talk Between Oxidative Stress and Inflammasome Pathway

Claudio Pirozzi ^1^, Adriano Lama ^1^, Chiara Annunziata ^1^, Gina Cavaliere ^2^,
Clara Ruiz Fernandez ^1,3^, Anna Monnolo ^4^, Federica Comella ^1^, Oreste Gualillo ^3^,
Mariano Stornaiuolo ^1^, Maria Pina Mollica ^2^, Giuseppina Mattace Raso ^1^,
Maria Carmela Ferrante ^4,^*^,†^ and Rosaria Meli ^1,^*^,†^

^1^ Department of Pharmacy, University of Naples Federico II, Via Domenico Montesano 49, 80131 Naples, Italy; claudio.pirozzi@unina.it (C.P.); adriano.lama@unina.it (A.L.); chiara.annunziata@unina.it (C.A.); clararf94@gmail.com (C.R.F.); federica.comella@unina.it (F.C.); mariano.stornaiuolo@unina.it (M.S.); mattace@unina.it (G.M.R.)

^2^ Department of Biology, University of Naples Federico II, Cupa Nuova Cinthia 21-Edificio 7,
80126 Naples, Italy; gina.cavaliere@unina.it (G.C.); mpmollic@unina.it (M.P.M.)

^3^ SERGAS (Servizo Galego de Saude) and IDIS (Instituto de Investigación Sanitaria de Santiago),
The NEIRID Lab (Neuroendocrine Interactions in Rheumatology and Inflammatory Diseases),
Research Laboratory 9, Santiago University Clinical Hospital, 15706 Santiago de Compostela, Spain; oreste.gualillo@sergas.es

^4^ Department of Veterinary Medicine and Animal Production, University of Naples Federico II,
Via Delpino 1, 80137 Naples, Italy; anna.monnolo@unina.it

***** Correspondence: ferrante@unina.it (M.C.F.); meli@unina.it (R.M)

† These authors are listed as co-seniors.

Table of Contents

[1. Supplementary Material and methods 2](#_Toc50128694)

[2. Supplementary Results 3](#_Toc50128695)

[References 4](#_Toc50128696)

**1. Supplementary Materials and methods**

*1.1. DNA methylation analysis*

Genomic DNA extraction from liver samples was performed with QIAamp Fast DNA Tissue Kit (Qiagen, Hilden, Germany). The quantity and purity of DNA were determined by a NanoDrop spectrophotometer. DNA was analysed by Liquid Chromatography-Mass Spectrometry (LC-MS) as previously described [1]. Isolated genomic DNA samples (1 μg) were enzymatically hydrolysed to individual deoxyribonucleosides by DNAse, phosphodiesterase I, alkaline phosphatase (all from BioLabs) in 50 mM Tris-HCl buffer pH 7.2 at 37°C for 16 h. Global DNA methylation and hydroxy-methylation were obtained by quantifying 5 methyl-deoxy-Cytosine (5mdC), 5 hydroxy-methyl-deoxy-Cytosine, and deoxy-Cytosine using LC/MS. The HPLC system Jasco Extrema LC-4000 system (Jasco Inc., Ithaca, NY) was coupled to an Advion Expression mass spectrometer (Advion Inc., Ithaca, NY) equipped with an ESI source. The mobile phase used for the chromatographic separation was a mixture of 0.1% formic acid in water and 0.1% formic acid in acetonitrile. The analyses were performed in the positive ESI mode. Six replicates were run for each sample. Global DNA methylation is expressed as a percentage of 5mdC versus the sum of 5mdC, 5 hydroxy-methyl-deoxy-Cytosine, and deoxy-Cytosine.

*1.2. Statistical analysis*

Data are presented as the mean ± SEM unless otherwise indicated. Differences among experimental groups were investigated through the one-way analysis of variance (ANOVA) for multiple comparisons followed by Bonferroni’s post hoc test, using GraphPad Prism 8 (GraphPad Software, San Diego, CA, USA). Statistical significance was set at P< 0.05 in all the statistical analyses.

**2. Supplementary results**

*2.1 BPA induces genomic DNA hypermethylation in obese mice*

In STD fed mice, BPA has been already associated with reduced deoxy-cytosine methylation [1,2]. However, in mice fed with either HFD or STD enriched in Methyl donors, i.e. Methionine or Sulpho Adenosyl Methionine (SAM), BPA has been shown to revert and cause DNA hypermethylation [3,4].

To identify gross epigenetic changes induced by BPA in HFD mice, we performed a global measurement of cytosine methylation by LC-MS. Despite this experimental approach is not suitable for determining the precise genomic location of methylation, it represents an ideal analytical tool for the measurement of environmental- and dietary-induced global epigenetic modifications. As shown in Figure S1, we measured a significant increase in deoxy-cytosine methylation in HFD+BPA group when compared with the HFD and STD, evidencing BPA capability to increase global DNA hypermethylation induced in mice by HFD.

**Figure S1.** BPA increases hepatic global DNA methylation induced by HFD. Global DNA methylation measured in genomic DNA extracted from liver samples of STD, HFD or HFD+BPA mice. DNA methylation is expressed as a percentage of 5 methyl-deoxy-cytosine (% 5mdC), *vs* the sum of 5mdC, 5 hydroxy-methyl-deoxy-cytosine, and deoxy-cytosine measured by LC/MS. 5mdC levels were significantly higher in BPA-treated mice than HFD and STD (n=6) (***P<0.001vs STD; #### P<0.0001 vs HFD+BPA).

**References**

1. Tiffon, C. The Impact of Nutrition and Environmental Epigenetics on Human Health and Disease. *Int J Mol Sci* **2018**, *19*, doi:10.3390/ijms19113425.

2. Jang, H.; Serra, C. Nutrition, epigenetics, and diseases. *Clin Nutr Res* **2014**, *3*, 1-8, doi:10.7762/cnr.2014.3.1.1.

3. Dolinoy, D.C.; Huang, D.; Jirtle, R.L. Maternal nutrient supplementation counteracts bisphenol A-induced DNA hypomethylation in early development. *Proc Natl Acad Sci U S A* **2007**, *104*, 13056-13061, doi:10.1073/pnas.0703739104.

4. Leung, Y.K.; Govindarajah, V.; Cheong, A.; Veevers, J.; Song, D.; Gear, R.; Zhu, X.; Ying, J.; Kendler, A.; Medvedovic, M., et al. Gestational high-fat diet and bisphenol A exposure heightens mammary cancer risk. *Endocr Relat Cancer* **2017**, *24*, 365-378, doi:10.1530/ERC-17-0006.
