# Supplementary figures and images for: Oral Bisphenol A Worsens Liver Immune-Metabolic and Mitochondrial Dysfunction Induced by High-Fat Diet in Adult Mice: Cross-Talk between Oxidative Stress and Inflammasome Pathway
Source: Antioxidants (Basel). 2020 Nov 30;9(12):1201. doi: 10.3390/antiox9121201 (PMC7760359; doi:10.3390/antiox9121201)

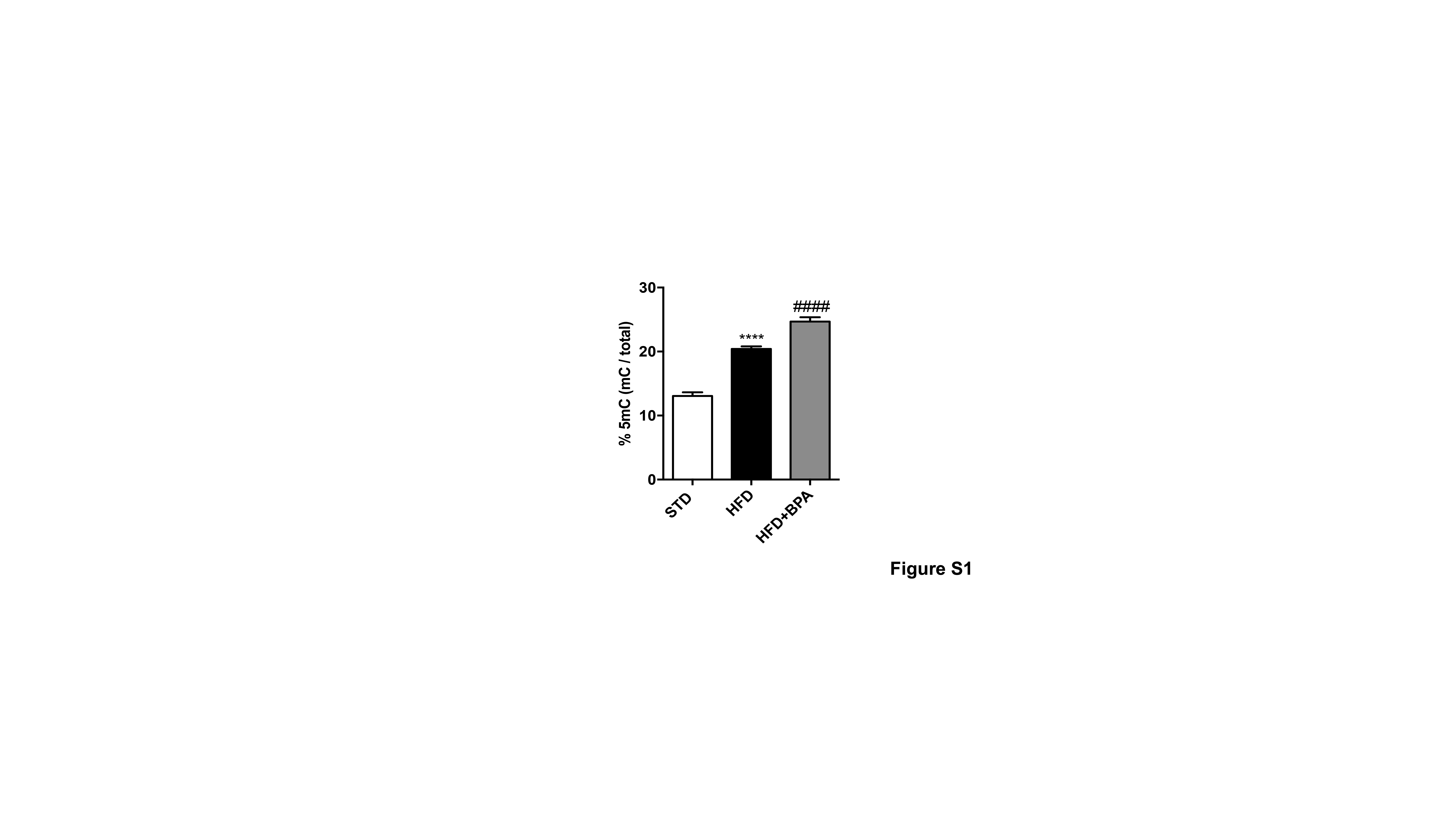

Supplement: Supplementary file 1 [file antioxidants-09-01201-s001.zip › antioxidants-1010380-suppl-final check/Figure S1.tif]
